# Supplementary material for: Hot spot prediction in protein-protein interactions by an ensemble system
Source: BMC Syst Biol. 2018 Dec 31;12(Suppl 9):132. doi: 10.1186/s12918-018-0665-8 (PMC6311905; doi:10.1186/s12918-018-0665-8)
Supplement: Supplementary file 2 — All relevant MATLAB code. A simple Matlab implement of our predictor. (ZIP 19,742 kb) [file 12918_2018_665_MOESM2_ESM.zip › Additional file 2/Read ASA/ASA website/SKEMPI/1PPFI.htm]

 NetSurfP - Protein Surface Accessibility and Secondary Structure Predictions

  

|  |  |  |
| --- | --- | --- |
|  |  | NetSurfP - Protein Surface Accessibility and Secondary Structure Predictions  Technical University of Denmark |

  


---

```
# For publication of results, please cite:
# A generic method for assignment of reliability scores applied to solvent accessibility predictions.
# Bent Petersen, Thomas Nordahl Petersen, Pernille Andersen, Morten Nielsen and Claus Lundegaard
# BMC Structural Biology 2009, 9:51 doi:10.1186/1472-6807-9-51
#
# Column 1: Class assignment - B for buried or E for Exposed - Threshold: 25% exposure, but not based on RSA
# Column 2: Amino acid
# Column 3: Sequence name
# Column 4: Amino acid number
# Column 5: Relative Surface Accessibility - RSA
# Column 6: Absolute Surface Accessibility
# Column 7: Z-fit score for RSA prediction
# Column 8: Probability for Alpha-Helix
# Column 9: Probability for Beta-strand
# Column 10: Probability for Coil
E L  1PPFI                  1    0.708 129.616  -1.774   0.003   0.003   0.994
E A  1PPFI                  2    0.556  61.238  -0.539   0.018   0.088   0.893
E A  1PPFI                  3    0.563  62.065  -0.177   0.021   0.279   0.699
E V  1PPFI                  4    0.374  57.545   0.038   0.022   0.359   0.619
E S  1PPFI                  5    0.602  70.543   1.022   0.022   0.359   0.619
B V  1PPFI                  6    0.224  34.490   0.249   0.022   0.359   0.619
E D  1PPFI                  7    0.558  80.422   0.511   0.021   0.279   0.699
B C  1PPFI                  8    0.063   8.887  -1.139   0.052   0.084   0.864
E S  1PPFI                  9    0.712  83.446   0.787   0.053   0.043   0.903
E E  1PPFI                 10    0.607 106.078   0.632   0.053   0.043   0.903
B Y  1PPFI                 11    0.206  43.937   0.800   0.053   0.043   0.903
E P  1PPFI                 12    0.559  79.365   0.202   0.018   0.047   0.935
E K  1PPFI                 13    0.597 122.823  -0.083   0.018   0.088   0.893
E P  1PPFI                 14    0.349  49.523  -0.117   0.018   0.088   0.893
E A  1PPFI                 15    0.368  40.576  -0.574   0.018   0.047   0.935
B C  1PPFI                 16    0.048   6.753  -0.380   0.018   0.019   0.964
E T  1PPFI                 17    0.364  50.501  -0.167   0.018   0.047   0.935
E L  1PPFI                 18    0.417  76.408  -0.651   0.052   0.084   0.864
E E  1PPFI                 19    0.455  79.454  -0.461   0.056   0.142   0.802
B Y  1PPFI                 20    0.218  46.501   0.032   0.056   0.142   0.802
E R  1PPFI                 21    0.400  91.669  -0.298   0.052   0.084   0.864
B P  1PPFI                 22    0.130  18.404  -0.476   0.052   0.084   0.864
B L  1PPFI                 23    0.107  19.500   0.034   0.064   0.216   0.721
B C  1PPFI                 24    0.058   8.199   0.191   0.066   0.296   0.638
B G  1PPFI                 25    0.176  13.820  -1.194   0.056   0.142   0.802
E S  1PPFI                 26    0.433  50.771  -1.012   0.053   0.043   0.903
E D  1PPFI                 27    0.483  69.629  -0.581   0.018   0.019   0.964
E N  1PPFI                 28    0.408  59.717  -1.667   0.018   0.019   0.964
E K  1PPFI                 29    0.393  80.922   0.260   0.018   0.088   0.893
E T  1PPFI                 30    0.311  43.094  -0.582   0.064   0.216   0.721
B Y  1PPFI                 31    0.162  34.641   0.721   0.113   0.087   0.800
E G  1PPFI                 32    0.536  42.167  -0.878   0.181   0.016   0.803
B N  1PPFI                 33    0.232  33.979  -1.127   0.430   0.016   0.555
E K  1PPFI                 34    0.378  77.775   0.679   0.751   0.050   0.199
B C  1PPFI                 35    0.050   7.062   0.407   0.779   0.100   0.120
E N  1PPFI                 36    0.306  44.813   0.955   0.779   0.100   0.120
B F  1PPFI                 37    0.095  19.046   0.479   0.807   0.137   0.056
B C  1PPFI                 38    0.099  13.872  -0.215   0.870   0.077   0.053
B N  1PPFI                 39    0.281  41.138   0.091   0.870   0.077   0.053
E A  1PPFI                 40    0.338  37.193   0.011   0.779   0.100   0.120
B V  1PPFI                 41    0.264  40.592   0.546   0.725   0.163   0.112
B V  1PPFI                 42    0.173  26.605   0.420   0.725   0.163   0.112
E E  1PPFI                 43    0.600 104.837   0.358   0.538   0.173   0.289
E S  1PPFI                 44    0.573  67.156  -0.997   0.191   0.086   0.723
E N  1PPFI                 45    0.669  97.942   0.020   0.018   0.019   0.964
E G  1PPFI                 46    0.403  31.677  -0.497   0.005   0.045   0.951
E T  1PPFI                 47    0.483  67.006   1.198   0.005   0.262   0.733
B L  1PPFI                 48    0.041   7.452  -0.141   0.003   0.718   0.279
E T  1PPFI                 49    0.354  49.114   0.984   0.001   0.900   0.099
B L  1PPFI                 50    0.114  20.965   0.721   0.001   0.900   0.099
E S  1PPFI                 51    0.381  44.618   0.959   0.001   0.900   0.099
E H  1PPFI                 52    0.293  53.224  -0.206   0.002   0.816   0.182
E F  1PPFI                 53    0.533 107.033   0.019   0.004   0.420   0.576
E G  1PPFI                 54    0.297  23.366  -2.609   0.005   0.262   0.733
E K  1PPFI                 55    0.669 137.634   1.289   0.004   0.197   0.799
E C  1PPFI                 56    0.507  71.183  -1.954   0.003   0.003   0.994

Explain the output.  Go back.
```

---
